# Caterpillars induce jasmonates in flowers and alter plant responses to a second attacker

**DOI:** 10.1111/nph.14904

**Published:** 2017-12-05

**Authors:** Lucille T. S. Chrétien, Anja David, Eirini Daikou, Wilhelm Boland, Jonathan Gershenzon, David Giron, Marcel Dicke, Dani Lucas‐Barbosa

**Affiliations:** ^1^ Laboratory of Entomology Wageningen University Droevendaalsesteeg 1, Radix building 6708PB Wageningen the Netherlands; ^2^ Institut de Recherche sur la Biologie de l'Insecte (IRBI) UMR 7261 CNRS/Université François‐Rabelais de Tours Avenue Monge, Parc Grandmont 37200 Tours France; ^3^ Department of Biology École Normale Supérieure de Lyon (ENS L) 46 Allée d'Italie 69007 Lyon France; ^4^ Department of Bioorganic Chemistry Max Planck Institute for Chemical Ecology (MPI CE) Beutenberg Campus, Hans‐Knoell‐Strasse 8 D‐07745 Jena Germany; ^5^ Department of Biochemistry Max Planck Institute for Chemical Ecology (MPI CE) Beutenberg Campus, Hans‐Knoell‐Strasse 8 D‐07745 Jena Germany

**Keywords:** *Brassica nigra* (Brassicaceae), florivorous insects, flowers, multiple attack, parasitoids, phytohormones, phytopathogens, plant resistance

## Abstract

- In nature, herbivorous insects and plant pathogens are generally abundant when plants are flowering. Thus, plants face a diversity of attackers during their reproductive phase. Plant responses to one attacker can interfere with responses to a second attacker, and phytohormones that orchestrate plant reproduction are also involved in resistance to insect and pathogen attack. We quantified phytohormonal responses of flowering plants exposed to single or dual attack and studied resistance mechanisms of plants in the flowering stage.
- Flowering *Brassica nigra* were exposed to either a chewing caterpillar, a phloem‐feeding aphid or a bacterial pathogen, and plant hormonal responses were compared with dual attack situations. We quantified phytohormones in inflorescences and leaves, and determined the consequences of hormonal changes for components of direct and indirect plant resistance.
- Caterpillars were the main inducers of jasmonates in inflorescences, and the phytohormonal profile of leaves was not affected by either insect or pathogen attack. Dual attack increased plant resistance to caterpillars, but compromised resistance to aphids. Parasitoid performance was negatively correlated with the performance of their hosts.
- We conclude that plants prioritize resistance of reproductive tissues over vegetative tissues, and that a chewing herbivore species is the main driver of responses in flowering *B. nigra*.

## Introduction

During their life time, plants interact with a multitude of organisms, and plant attackers are generally abundant during the flowering period (Lucas‐Barbosa *et al*., 2014; Schlinkert *et al*., 2015). Plants evolved various defense strategies to defend against a multitude of attackers and to maximize their fitness (Dicke & Hilker, 2003; Howe & Jander, 2008; Agrawal, 2011; Karban, 2011; Dicke & van Loon, 2014). Plant resistance traits can be induced upon attack and directly affect the performance and survival of plant antagonists or enhance the effectiveness of natural enemies of the plant attackers (Dicke & Hilker, 2003; Dicke & Baldwin, 2010; Wu & Baldwin, 2010). Inducible resistance traits of plants can vary depending on the ontogenetic stage of the plant (Barton & Koricheva, 2010; Erbilgin & Colgan, 2012; Quintero *et al*., 2014), on the identity of the attacker (Erb *et al*., 2012; Dicke & van Loon, 2014) and on whether the plant is attacked by a single or by multiple species (Soler *et al*., 2012; Kroes *et al*., 2015). Such specificity in the induction and regulation of plant responses to attack allows plants to activate resistance traits specifically in targeted tissues and to mount tailor‐made resistance to different attackers (Pieterse & Dicke, 2007; Karban, 2011; Mithöfer & Boland, 2012).

A few phytohormones regulate the main biosynthetic pathways in plants, and these can play a role in adjusting plant defense strategies to different attackers (Heidel & Baldwin, 2004; Erb *et al*., 2012). Jasmonic acid (JA) is the main phytohormone involved in plant responses to chewing herbivores and necrotrophic pathogens, whereas salicylic acid (SA) is the main phytohormone mediating plant responses to phloem‐feeding herbivores and biotrophic pathogens (Heidel & Baldwin, 2004; Wu & Baldwin, 2010; Lazebnik *et al*., 2014). Other phytohormones such as abscisic acid (ABA) and cytokinins (CKs) seem to be more specific, as they accumulate particularly in response to certain species of chewing herbivores and pathogens (Bari & Jones, 2009; Ton *et al*., 2009). In nature, plants are often simultaneously or successively challenged by multiple attackers, and the synergistic or antagonistic nature of phytohormonal responses can shape a plant's phenotype and determine plant resistance or susceptibility to multiple attackers (Koornneef & Pieterse, 2008; Lazebnik *et al*., 2014).

When plants are challenged by attackers from different feeding guilds, the induction of distinct phytohormones can have antagonistic effects due to negative crosstalk between signaling pathways. Indeed, although exceptions occur, SA and JA usually have antagonistic effects (Koornneef & Pieterse, 2008; Erb *et al*., 2012; Thaler *et al*., 2012), and this can modulate the expression of plant resistance. Plant indirect resistance can also be influenced by plant responses to multiple attack. Changes in herbivore performance can positively or negatively affect the attraction and performance of their natural enemies (Henry *et al*., 2005; Rodriguez‐Saona *et al*., 2005; Kos *et al*., 2012; Soler *et al*., 2012). Therefore, a plant's response to one attacker can interfere with the response to another attacker, and consequently positively or negatively impact both direct and indirect plant resistance.

To date, the chemical and ecological consequences of plant responses to multiple attack have been exclusively studied for plants in the vegetative stage, although resistance of plants in the flowering stage is directly linked to plant fitness. The same phytohormones that mediate resistance to insects and pathogens also influence plant reproduction (Santner & Estelle, 2009; Avanci *et al*., 2010; Giron *et al*., 2013; Santino *et al*., 2013). For instance, SA is involved in the induction of flowering (Martínez *et al*., 2004; Wada & Takeno, 2010; Rivas‐San Vicente & Plasencia, 2011). JA is essential for male fertility (Stintzi & Browse, 2000; Wasternack & Hause, 2013) and petal growth (Brioudes *et al*., 2009), and affects the allocation of resources between different organs (Babst *et al*., 2005). ABA is involved in pod abscission (Liu *et al*., 2003) and may induce bud formation and flowering (Samuoliene *et al*., 2009). The induction of phytohormones by attackers could thus interfere with the regulation of plant reproduction. Consequently, we expect plants that are attacked in the flowering stage and plants attacked in the vegetative stage to have different profiles of phytohormones. Moreover, recent studies have shown that herbivore attack to plants in the flowering stage induces primary and secondary metabolic changes in flowers, rather than in leaves (Pareja *et al*., 2012; Bruinsma *et al*., 2014; Lucas‐Barbosa, 2016). Such results suggest that plants can differentially allocate resources to leaves or inflorescences, as well as activate resistance traits specifically in flower or leaf tissues. Despite the evidence that herbivore attack to leaves and flowers influences the metabolic profile of flowers (Pareja *et al*., 2012; Bruinsma *et al*., 2014; Lucas‐Barbosa, 2016), to our knowledge no studies have investigated how plants in the flowering stage deal with multiple attack on flowers, nor what the consequences are for plant hormonal regulation of resistance and reproductive processes.

Here, we investigated phytohormonal responses of flowering plants to single or dual attack, by two insect species and a bacterial pathogen. We expected to detect higher resistance levels in flowers than in leaves, and that the plant phytohormonal profile is characteristic of the type of attacker and combination of attackers. To investigate these questions, we quantified phytohormone concentrations in leaves and inflorescences of plants exposed to single or dual attack, and compared this with concentrations in plant tissues of non‐exposed control plants. We investigated how phytohormonal responses to single or dual attack are reflected in plant resistance to insects, as well as the cascading effects on the natural enemies of the herbivores.

## Materials and Methods

### Study system

Black mustard *Brassica nigra* L. (Brassicales: Brassicaceae) is an annual plant, generally considered to be an outcrossing species (Conner & Neumeier, 1995) although some selfing can occur (Lucas‐Barbosa *et al*., 2013, 2017). In nature, *B. nigra* is attacked by specialist herbivores such as the cabbage aphid *Brevicoryne brassicae* L. (Hemiptera: Aphididae) and the large cabbage white butterfly *Pieris brassicae* L. (Lepidoptera: Pieridae), as well as pathogens such as the bacterium *Xanthomonas campestris* pathovar *raphani* (Xcr). This bacterium is the agent of the leaf spot disease that forms small necrotic spots (*c*. 1–3 mm) on leaves of many Brassicaceae, but rarely kills the plants (Machmud, 1982; Vicente *et al*., 2006). The two insect attackers can damage flowers of brassicaceous plants (Lucas‐Barbosa *et al*., 2013; L. T. S. Chrétien, pers. obs.), and Xcr can spread from infected leaves to mature seeds of broccoli plants (Machmud, 1982). These three attackers are expected to induce distinct responses in *B. nigra*. The phloem‐feeding aphid *B. brassicae* is expected to mainly induce the SA pathway (Mewis *et al*., 2005; Koornneef & Pieterse, 2008; Erb *et al*., 2012). Caterpillars of *P. brassicae* are chewing herbivores, which generally induce the JA/ethylene (ET) pathway as well as ABA (Mewis *et al*., 2005; Koornneef & Pieterse, 2008; Erb *et al*., 2012; Vos *et al*., 2013). Xcr can induce the production of JA and SA (Bonnet *et al*., 2017), and ET mediates resistance against Xcr (Ton *et al*., 2002). Both insect herbivores, *B. brassicae* and *P. brassicae*, are frequently attacked by parasitic wasps. The solitary parasitoid *Diaeretiella rapae* McIntosh (Hymenoptera: Braconidae) is the main parasitoid of *B. brassicae* in the Netherlands (Hafez, 1961), and parasitizes aphids associated with Brassicaceae (Bahana & Karuhize, 1986; Vaughn *et al*., 1996). *Cotesia glomerata* L. (Hymenoptera: Braconidae) is a gregarious specialist parasitoid and the main parasitoid of *P. brassicae* (Geervliet & Brodeur, 1992; Brodeur *et al*., 1998).

### Plant, insect and bacteria cultures

We used a mixture of seeds from at least 20 individual *B. nigra* plants that had been exposed to open pollination in a field of the experimental farm of Wageningen University, the Netherlands (Lucas‐Barbosa *et al*., 2013). Plants grew in pots (Ø17 cm, 2 litres) filled with a mixture of potting soil and sand (1 : 1, v/v), in a glasshouse compartment (22 ± 2°C, 50–70% relative humidity (RH), 16 h : 8 h, light : dark).

*B. brassicae* aphids were reared on Brussels sprout (*Brassica oleracea* var. *gemmifera*) plants in a glasshouse compartment (21 ± 1°C, 50–70% RH, 16 h : 8 h, light : dark). The parasitic wasp *D. rapae* was reared on *B. brassicae* aphids on Brussels sprout plants in a climate cabinet (25 ± 1°C, 16 h : 8 h, light : dark). Honey from organic production and water were provided to the adult wasps.

*P. brassicae* caterpillars were also reared on Brussels sprout plants in a climate room (21 ± 1°C, 50–70% RH, 16 h : 8 h, light : dark), and pupae and adult *P. brassicae* were kept in a glasshouse compartment (25 ± 1°C, 50–70% RH, 16 h : 8 h, light : dark). Butterflies fed on honey solution (10% w/v) from organic production. To rear *C. glomerata*, neonate caterpillars were parasitized by *C. glomerata* and reared on Brussels sprout plants in a climate room (21 ± 1°C, 50–70% RH, 16 h : 8 h, light : dark). Adult wasps were kept in a climate cabinet (25 ± 1°C, 16 h : 8 h, light : dark) and provided with honey from organic production and water.

Xcr was obtained from Utrecht University, the Netherlands (Ponzio *et al*., 2014). Xcr was cultured in an artificial liquid medium nutrient broth (8 g l^−1^ (Difco): beef extract 3.0 g l^−1^ and peptone 5.0 g l^−1^; BD Diagnostics, Franklin Lakes, NJ, USA) for *c*. 22 h at 28°C and shaken at 160 rpm. Cells of Xcr were obtained by centrifuging the culture broth twice at 3000 relative centrifugal force for 10 min and re‐suspending the pellet containing the bacterial cells in buffer (MgSO_4_, 10 mM) after each centrifugation. We estimated the concentration of the final inoculum (10^9^ cells ml^−1^) by measuring the light absorbance at 600 nm.

### Plant treatment – induction of *B. nigra* plants by single or simultaneous dual attack

**Figure 1:**
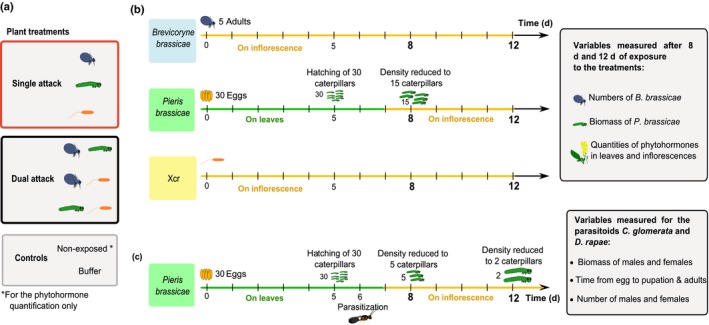
Schematic representation of the treatments applied to *Brassica nigra* plants and timeline of the experiments. (a) Description of the treatments. Flowering *B. nigra* were exposed to one attacker, exposed simultaneously to two attackers, to buffer only (control) or were non‐exposed. Plant treatments that did not require bacterial infection had a floral leaf exposed to buffer solution instead of the inoculum containing the bacterium. (b) Timeline of the experiments with herbivores and/or pathogens. Plants were exposed to one of the treatments for 8 and 12 d, and concentrations of phytohormones or herbivore performance were then assessed. Caterpillar density was reduced by 50% on day 8, to mimic natural dispersal to neighboring plants. (c) Timeline of the experiments with parasitoids. L_1_ caterpillars were parasitized on day 6 by parasitoid wasps, and caterpillar density was reduced from 30 caterpillars to five at 8 d after infestation, and from five to two at 12 d after infestation.

Within 2 d after opening of the first flowers, *B. nigra* plants were exposed to one or two attackers, or kept as control. Plants were exposed to a single attacker, either *B. brassicae*,* P. brassicae* or Xcr, or simultaneously exposed to two of these three attackers. Control plants were exposed to buffer only, or kept untreated (Fig. 1a). We exposed experimental plants to densities of insect attackers commonly observed in the field to set ecologically relevant conditions (Lucas‐Barbosa *et al*., 2013, 2014; D. Lucas‐Barbosa and L. T. S. Chrétien, pers. obs.). To infest *B. nigra* with *B. brassicae* (Fig. 1b), we gently placed five young adult females on a bract (flower leaf), at the base of the inflorescence. Shortly after infestation, the aphids moved to the flower stems where they quickly established large colonies by asexual reproduction. It is common to observe an early infestation of *B. nigra* flowers by one to 10 *B. brassicae* adults in the field (D. Lucas‐Barbosa & L. T. S. Chrétien, pers. obs.). *P. brassicae* lay eggs in clutches on the leaves of flowering *B. nigra* (Lucas‐Barbosa *et al*., 2014) and, after hatching, L_1_ or L_2_ caterpillars move to the inflorescence and become florivores (Lucas‐Barbosa *et al*., 2013). To infest *B. nigra* with *P. brassicae* (Fig. 1b), plants were exposed to butterflies until a clutch of at least 30 eggs was laid on a leaf, and any extra eggs were gently removed with forceps. A fine mesh covered the inflorescence to protect open flowers from pollination by the butterflies while plants were exposed to them. *P. brassicae* caterpillars hatched from the eggs at 5 d after oviposition (Fig. 1b). The newly hatched caterpillars fed transiently on leaves (*c*. 2 d), and generally moved to the flowers, at 6–7 d after oviposition. When caterpillars had not moved, they were transferred to the flowers at 7 d after oviposition (Fig. 1b) to ensure damage to flowers for at least 24 h before the first plant sampling and measurements on day 8. Eight days after infestation, caterpillar density was reduced by 50% to mimic natural predation and dispersal to neighboring *B. nigra* plants as observed in the field, and to prevent complete consumption of flowers (Fig. 1b). Caterpillar survival was not affected by any of the treatments. For infestation with Xcr (Fig. 1b), 500 μl of the bacterium inoculum (10^9^ cells ml^−1^ in buffer) was applied on the underside of a bract, at the base of the inflorescence. A soft‐clip was used to keep a piece of cotton wool (2 × 2 cm) containing the inoculum attached to the bract for 4 h as Xcr enters plant tissues via stomata (McCulloch, 1929; Machmud, 1982). The described methodology was adapted from techniques commonly used, which consist of either spraying the plant with inoculum (Machmud, 1982; Vicente *et al*., 2006), applying the inoculum with cotton wool (McCulloch, 1929) or dipping the plant part in inoculum (De Vos *et al*., 2006). For the experimental plants that were used for phytohormone quantification, we recorded necrotic spots that could either represent the plant hypersensitive response (HR) or a disease symptom. Mustard plants are relatively resistant to Xcr and the disease rarely spreads throughout the plant (McCulloch, 1929; Vicente *et al*., 2006; Ponzio, 2016; Ponzio *et al*., 2016b). For recordings at day 12, necrotic spots were observed on 50% of the plants per treatment, and for recordings at day 8, necrotic spots were observed on 33–50% of the plants per treatment. To control for a possible effect of the buffer on plant responses, plants exposed to aphids or caterpillars only, or aphids plus caterpillars simultaneously, were clipped for 4 h with buffer solution containing no bacteria. In addition, two control treatments were added: plants that received no treatment, and plants that were clipped for 4 h with bacteria‐free buffer solution. Within a plant, a single bract never received more than one treatment. Exposed and control plants were kept in a glasshouse compartment (21 ± 1°C, 50–70% RH, 16 h : 8 h, light : dark) until sampling. Dual attack consisted of simultaneously exposing plants to two attackers (methods same as above).

### Sampling and quantification of phytohormones in leaves and inflorescences of *B. nigra* upon single and dual attack

To investigate the induction of phytohormonal responses in flowering *B. nigra* plants exposed to three types of single attackers or simultaneous exposure to two attackers, phytohormone concentrations were quantified in leaves and inflorescences of plants exposed to one of eight different treatments: *B. brassicae*,* P. brassicae*, Xcr, *B. brassicae* plus *P. brassicae*,* B. brassicae* plus Xcr, *P. brassicae* plus Xcr, buffer (control) and nontreated (control) (Fig. 1a). After 8 and 12 d of exposure to the treatments, we sampled leaves and inflorescences for the quantification of phytohormones. Shortly before harvesting, all insects were removed from the plants. All true leaves and inflorescences were harvested, immediately frozen in liquid nitrogen and kept at −80°C. True leaves and inflorescences were then freeze‐dried, ground and kept at −20°C. The bracts or leaves originally exposed to the insects or to the bacterial inoculum were not harvested. We focused on three key phytohormones, ABA, SA and JA, including precursors, active forms and degradation forms of JA . Thus, we quantified the concentration of the phytohormones ABA, SA, JA and the precursor of JA, *cis*‐(+)‐12‐oxophytodienoic acid (*cis*‐OPDA) (Heitz *et al*., 2016). In addition, we quantified (+)‐7‐iso‐jasmonoyl‐ʟ‐isoleucine ((+)‐7‐iso‐JA‐Ile) assumed to be the most active form of JA and (−)‐jasmonoyl‐ʟ‐isoleucine ((−)‐JA‐Ile), a less active form of JA (Fonseca *et al*., 2009; Avanci *et al*., 2010), and we quantified the degradation products of JA that are nonactive: 12‐hydroxy‐jasmonate (12‐OH‐JA), 12‐hydroxy‐jasmonoyl‐isoleucine (12‐OH‐JA‐Ile) and 12‐carboxy‐jasmonoyl‐isoleucine (12‐COOH‐JA‐Ile) (Heitz *et al*., 2016). Phytohormone concentrations (ng g^−1^ of dry mass) were quantified for six plant replicates per treatment, and per time point. Extraction of phytohormones and analyses were performed following the method of Almeida Trapp *et al*. (2014), and as described in Supporting Information Methods S1.

### Effects of dual attack on plant direct resistance to aphids and caterpillars

To investigate whether different induction profiles of phytohormones are reflected in plant direct resistance or susceptibility to herbivorous insects when exposed to single and dual attack, we assessed the performance of *B. brassicae* aphids and of *P. brassicae* caterpillars that fed on *B. nigra* plants exposed to single attack by the herbivores, or to simultaneous attack by another herbivore or the bacteria (Fig. 1b). The performance of *B. brassicae* was assessed on *B. nigra* plants exposed to each of the following three treatments: *B. brassicae*,* B. brassicae* plus *P. brassicae*, and *B. brassicae* plus Xcr. The performance of *P. brassicae* was assessed on *B. nigra* plants exposed to each of the following three treatments: *P. brassicae*,* P. brassicae* plus *B. brassicae*, and *P. brassicae* plus Xcr. After 8 and 12 d of exposure to treatments, the number of aphids and the fresh biomass of caterpillars were used as proxies of plant resistance. For this, aphids generated by the five initial young females were counted one by one for colonies smaller than 100 aphids, and for larger colonies, the number of aphids was estimated based on the count of 100 aphids. After 8 d of exposure to treatments, 50% of the caterpillars (*c*. 15 caterpillars per plant) were randomly selected, weighed individually and discarded. After 12 d of exposure to treatment, the remaining caterpillars were weighed (*c*. 15 caterpillars per plant), and both caterpillars and plants were discarded. We had seven to eight plant replicates per treatment.

### Effects of dual attack on parasitoid performance

The performance of the parasitoid *D. rapae* was assessed in aphid hosts on plants exposed to each of three treatments: *B. brassicae*,* B. brassicae* plus *P. brassicae*, and *B. brassicae* plus Xcr; the performance of the parasitoid *C. glomerata* was assessed in caterpillar hosts on *B. nigra* plants exposed to each of three treatments: *P. brassicae*,* P. brassicae* plus *B. brassicae*, and *P. brassicae* plus Xcr. Host herbivores were parasitized after 6 d of exposure of the plant to the attackers. Female wasps used for parasitization were 3–6 d old, nonexperienced (naïve) and mated. For parasitization, 15 young aphid nymphs (randomly selected) or 30 *P. brassicae* L_1_ caterpillars were exposed for 90 min to 12 wasps. In the field, *D. rapae* only oviposits in the late‐instar nymphs within the aphid colony (Hafez, 1961) and *C. glomerata* parasitizes L_1_ caterpillars (Mattiacci & Dicke, 1995) and generally oviposits in all caterpillars in a clutch. We assumed that all nymphs and caterpillars were parasitized, and placed them back on the plant to complete their development. Caterpillar density was reduced from 30 to five caterpillars at 2 d after parasitization (day 8). Six days after parasitization (day 12), only two randomly selected caterpillars were kept on the plant to ensure that there would be enough plant material for the caterpillars to feed (Fig. 1c); the other three caterpillars were discarded. When the first aphid mummies became visible, aphid‐infested flower stalks were cut, and we kept the flower stalk with humidified cotton wool around it in a mesh box. Fifth instar (L_5_) caterpillars were collected before egression of the parasitoid larvae, and individual caterpillars were placed in separate mesh boxes. Boxes with mummies or caterpillars were placed in a climate cabinet (25 ± 1°C, 16 h : 8 h, light : dark) until adult *D. rapae* and *C. glomerata* wasps emerged. Parasitoid performance was assessed by measuring development time (egg to adult), fresh biomass of male and female adult wasps, and number of male and female adult wasps. To determine the developmental time of *D. rapae*, we recorded the date when the first mummies were observed (pupation of the wasp larvae) and the date of emergence of the first adults. To determine the developmental time of *C. glomerata* we recorded the date when the first pupal cocoons were observed, and the date of emergence of the first adults. Adult parasitoids were sexed and counted on the day they emerged from the mummies or cocoons, and stored at −20°C until they were individually weighed. For *D. rapae*, we had 15 parasitized aphids per plant and four to six plant replicates per treatment. The biomass of males and females that emerged from parasitized *B. brassicae* feeding on an individual plant was used for statistical tests. For *C. glomerata*, dozens of male and female wasps emerged per caterpillar, and we had two caterpillars per plant, and six to 10 plants per treatment. The mean biomass of female wasps and male wasps emerging per caterpillar was calculated and used for statistical tests on a per‐plant basis.

### Statistical analyses

Phytohormone profiles of different plant tissues and of plants subjected to different treatments were analysed by multivariate data analysis, using projection to latent structures discriminant analysis (PLS‐DA) with Umetrics SIMCA (Umetrics AB, released 2015, Version 14.0, Umeå, Sweden). Data for nontreated plants were not included in the discriminant analyses because phytohormone concentrations were similar to those in plants treated with buffer (Figs S1, S2; Tables S1, S2). We used a generalized linear model (GLM) with a likelihood ratio and chi‐square test to assess whether there was an effect of treatment, plant part or time point on the concentration of each of the phytohormones (overall), and whether there was an effect of treatment or plant part at each time point separately (day 8, day 12) on the concentrations of each of the phytohormones. We included treatment, time point and plant part as main factors plus all interactions in the first case, and treatment and plant part as main factors, and their interaction, in the second case. When a significant effect of one of the main factors or of an interaction was detected, a Bonferroni *post‐hoc* test was used to test for differences between treatments (overall effect), plant parts (leaves and inflorescences), and between each combination of treatment and plant part. We based the model on a normal distribution and Identity was specified as the link function in the model.

Experimental data on the development time, biomass and numbers of insects were also analysed by a GLM with a likelihood ratio and chi‐square test. We included in the model as main factors: treatment and time point when analyzing number of aphids and biomass of caterpillars; plant treatment and sex, when analyzing data related to biomass and numbers of parasitoids; and treatment and developmental stage when analyzing data related to the development time of the parasitoids. In all cases, interactions were included. Plant identity was nested within the factor treatment and included in the model. When a significant effect of one of the main factors was detected or when an interaction between factors was significant, a Bonferroni *post‐hoc* test was used to test for differences between treatments (overall effect), between the other main factors and between all combinations of factor levels. Data on insect biomass were analyzed by a GLM that was based on a normal distribution and the function Identity was specified as the link function in the model. The mean biomass of female or male *C. glomerata* wasps that emerged per caterpillar was used for the analysis. Data on insect numbers were assumed to follow a Poisson distribution, a quasi‐likelihood function was used to correct for overdispersion and Log was specified as the link function in the model. Data related to the developmental time of the parasitoids were first log‐transformed to meet assumptions of normality.

## Results

### Phytohormonal profile of leaves and inflorescences

**Figure 2:**
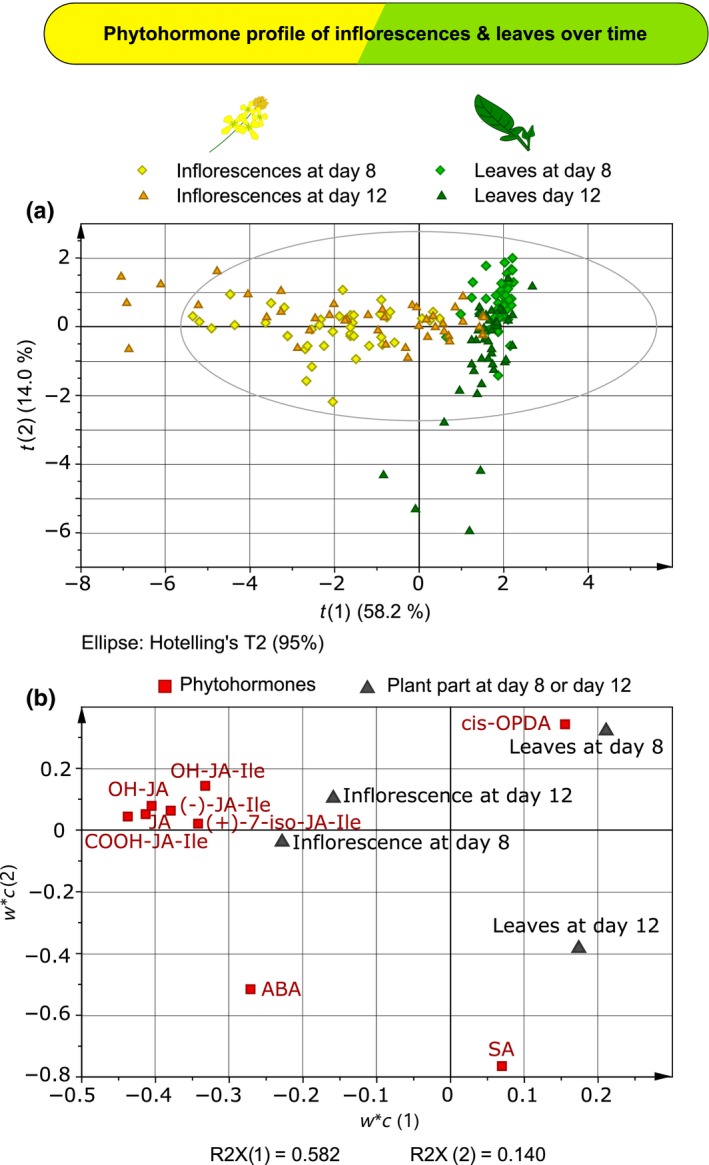
Phytohormonal profile of leaves and inflorescences of *Brassica nigra* exposed to single or dual attack for 8 or 12 d. Projection to latent structures discriminant analysis (PLS‐DA) of phytohormonal profile in inflorescences and leaves of *B. nigra* after 8 and 12 d of exposure to single or dual attack by *Brevicoryne brassicae*,* Pieris brassicae* and/or *Xanthomonas campestris* pv *raphani* (Xcr), or exposure to buffer (control). Six phytohormones were measured: salicylic acid (SA), abscisic acid (ABA), jasmonic acid (JA), *cis*‐(+)‐12‐oxophytodienoic acid (*cis*‐OPDA), (−)‐JA‐Ile and (+)‐7‐iso‐JA‐Ile; and three catabolites of JA: 12‐OH‐JA, 12‐OH‐JA‐Ile and 12‐COOH‐JA‐Ile. Phytohormone concentrations are expressed in ng g^−1^ of dry plant biomass. (a) Scatter plots show grouping pattern of samples from inflorescences at day 8, inflorescences at day 12, leaves at day 8 and leaves at day 12 according to the first two principal components. The Hotelling's ellipse confines the confidence region (95%) of the score plot. (b) Loading plots show the contribution of each of the phytohormone quantifications to the first two principal components.

We assessed plant responses to single and multiple attack by quantifying phytohormones in true leaves and inflorescences of plants that were either exposed to different individual attackers or combinations of attackers for 8 or 12 d, or treated with buffer (control). The first principal component of the PLS‐DA clearly separated leaf samples from those of inflorescences based on their phytohormonal profile; 58 and 14% of the total variance was explained by the first and second principal components, respectively (Fig. 2a). The jasmonates (JA, (+)‐7‐iso‐JA‐Ile, (−)‐JA‐Ile) and their catabolites (12‐OH‐JA, 12‐OH‐JA‐Ile, 12‐COOH‐JA‐Ile) as well as ABA were more abundant in inflorescences than in leaves, whereas SA and *cis*‐OPDA were more abundant in leaves than in inflorescences (Fig. 2b). Irrespective of the time points, the concentrations of jasmonates and their catabolites were 151–2242% higher in inflorescences than in leaves (Figs S1, S2; Tables S1, S2; GLM, overall, plant part, for (+)‐7‐iso‐JA‐Ile, (−)‐JA‐Ile, 12‐OH‐JA, 12‐OH‐JA‐Ile, 12‐COOH‐JA‐Ile and JA, *P *<* *0.001). Concentrations of ABA were 48% higher in inflorescences than in leaves (Fig. S1; Table S2, GLM, *P *<* *0.001). By contrast, concentrations of *cis*‐OPDA and SA were respectively 46 and 37% higher in leaves than in inflorescences (Fig. S1; Table S2; GLM, *cis*‐OPDA: *P *<* *0.001; SA: *P *=* *0.020).

Independent of attacker identity, time influenced the phytohormonal profile of the plants more strongly in leaves than in inflorescences (Fig. 2). SA concentration, for instance, was higher at day 12 than at day 8 in leaves but not in flowers (Fig. S1; Table S2; GLM, Bonferroni *post‐hoc* test, leaves day 8 vs day 12: *P *<* *0.001; inflorescence day 8 vs day 12: *P *=* *1.000). ABA concentration was also higher at day 12 than at day 8 in leaves but not in flowers (Fig. S2; Table S2; GLM, Bonferroni *post‐hoc* test, leaves day 8 vs day 12: *P *<* *0.001; inflorescence day 8 vs day 12: *P *=* *1.000). For the jasmonates, smaller temporal effects were recorded, and here the effects were detected in the inflorescences but not in the leaves. JA and (−)‐JA‐Ile concentration in inflorescences decreased slightly from 8 to 12 d, whereas the concentration of 12‐OH‐JA increased. JA concentration was 33% lower in inflorescences at day 12 than at day 8 (Fig. S2; Table S2; GLM Bonferroni *post‐hoc* test, inflorescence day 8 vs day 12: *P *<* *0.001; leaves day 8 vs day 12: *P *=* *1.000), and (−)‐JA‐Ile concentration was 15% lower in inflorescences at day 12 than at day 8 (Fig. S1; Table S1; GLM, Bonferroni *post‐hoc* test, inflorescence day 8 vs day 12: *P *=* *0.029; leaves day 8 vs day 12: *P *=* *1.000), whereas the concentration of OH‐JA‐Ile was 29% higher in inflorescences at day 12 than at day 8 (Fig. S1; Table S1; GLM, Bonferroni *post‐hoc* test, inflorescence day 8 vs day 12: *P *=* *0.016; leaves day 8 vs day 12: *P *=* *1.000). Time did not influence the concentration of (+)‐7‐iso‐JA‐Ile, 12‐OH‐JA, 12‐COOH‐JA‐Ile or *cis*‐OPDA (Figs S1, S2; Tables S1, S2; GLM, *P *>* *0.050).

### Phytohormonal profile of inflorescences of plants exposed to single and dual attack by insects and a pathogen

**Figure 3:**
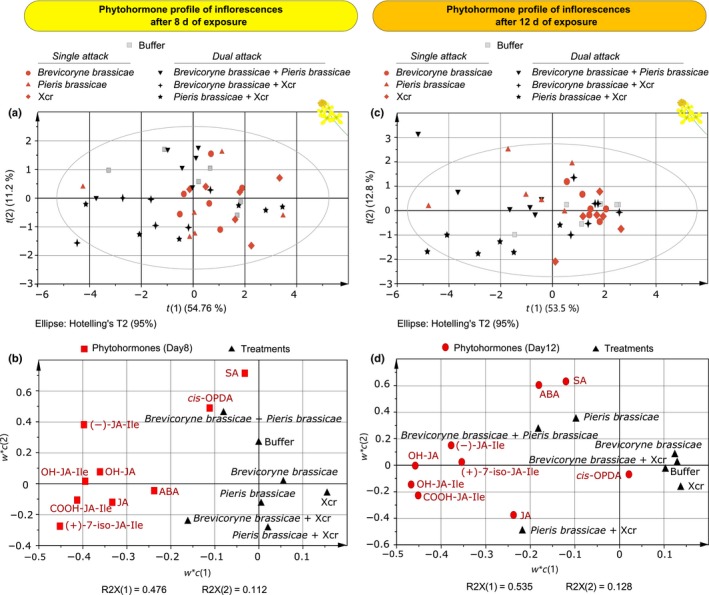
Phytohormonal profile of inflorescences of *Brassica nigra* exposed to single or dual attack for 8 and for 12 d. Projection to latent structures discriminant analysis (PLS‐DA) separating samples by treatment group for the phytohormonal response of inflorescences after 8 and 12 d of exposure of the plant to treatments. Six phytohormones were measured: salicylic acid (SA), abscisic acid (ABA), jasmonic acid (JA), *cis*‐(+)‐12‐oxophytodienoic acid (*cis*‐OPDA), (−)‐JA‐Ile and (+)‐7‐iso‐JA‐Ile; and three catabolites of JA: 12‐OH‐JA, 12‐OH‐JA‐Ile and 12‐COOH‐JA‐Ile. Phytohormone concentrations are expressed in ng g^−1^ of dry plant biomass. (a, c) Scatter plots show grouping pattern of samples from a single treatment according to the first two principal components. The Hotelling's ellipse confines the confidence region (95%) of the score plot. (b, d) Loading plots show the contribution of each of the phytohormone quantifications to the first two principal components.

Overall, phytohormone profiles of inflorescences were affected by exposure of plants to single and simultaneous dual attack, and particularly upon 12 d of exposure to the treatments (Fig. 3). The first principal component of the PLS‐DA clearly separated inflorescence samples of plants that had been exposed to single attack and dual attack involving caterpillars from inflorescence samples of plants that had not been exposed to caterpillars (Fig. 3c). Induction of biologically active jasmonates and their catabolites was affected by treatments that included *P. brassicae* caterpillars, either as single attackers or in combination with aphids or bacteria (Figs 3, S1). The second principal component separated samples of inflorescences that had been exposed to single attack from those exposed to dual attack; 54 and 13% of the total variance was explained by the first and second principal components, respectively. In particular, single attack by caterpillars and dual attack by caterpillars plus aphids were separated from samples of inflorescences that had been exposed to caterpillars plus bacteria for 12 d (Fig. 3c). Indeed, for the catabolites 12‐OH‐JA‐Ile and 12‐COOH‐JA‐Ile, concentrations were *c*. 50% higher in inflorescences exposed to caterpillars plus bacteria than in inflorescences exposed to caterpillars only (Fig. S1, GLM, Bonferroni *post‐hoc* test, caterpillar plus bacteria vs caterpillar, 12‐OH‐JA‐Ile: *P *=* *0.011; 12‐COOH‐JA‐Ile: *P *<* *0.001).

Overall, exposure of plants to either aphids or Xcr, or to dual attack by aphids plus Xcr, did not influence the phytohormonal profile of inflorescences, either at day 8 or at day 12 (Fig. 3). However, differences were present for some phytohormones (Fig. S1; Table S1). For instance, plants exposed to aphids plus bacteria had higher concentrations of (+)‐7‐iso‐JA‐Ile than plants exposed to either aphids only (*P *=* *0.002) or bacteria only (*P *=* *0.035).

Changes in the phytohormonal profile upon exposure of plants to attackers were tissue‐ and time‐specific. Flower attackers induced changes in the concentration of phytohormones in the inflorescences but not in the leaves (Figs S1, S2; Tables S1, S2). The effect of treatment on the phytohormonal profile was dependent on the time point, and most changes were observed after 12 d of exposure (Figs 3, S2; Tables S1, S2). After 8 d of exposure, treatments affected the concentration of one jasmonate, (+)‐7‐iso‐JA‐Ile, but after 12 d of exposure, treatments affected the concentration of five jasmonates, that is, (+)‐7‐iso‐JA‐Ile, (−)‐JA‐Ile, 12‐OH‐JA, 12‐OH‐JA‐Ile and 12‐COOH‐JA‐Ile (Figs S1, S2; Tables S1, S2).

### Effects of dual attack on plant direct resistance to aphids and caterpillars

**Figure 4:**
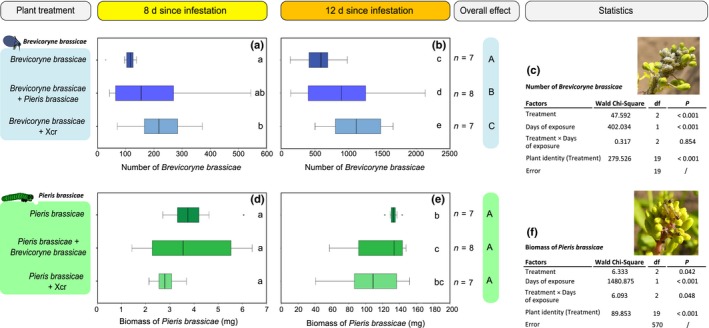
Number of *Brevicoryne brassicae* and fresh biomass of *Pieris brassicae* reared on flowering *Brassica nigra* plants exposed to single or dual attack. (a–c) Number of *B. brassicae* aphids (median, 1^st^ and 3^rd^ quartiles, ± SD) and (d–f) fresh biomass of *P. brassicae* caterpillars (median, 1^st^ and 3^rd^ quartiles, ± SD) determined after *B. nigra* plants had been exposed for (a, d) 8 d or (b, e) 12 d to single or dual attack by *B. brassicae*,* P. brassicae* and/or *Xanthomonas campestris* pv *raphani* (Xcr); (c, f) statistics. Overall effects of the treatment and days of exposure to treatments were tested with a general linear model with Poisson distribution (*B. brassicae* number) or normal distribution (*P. brassicae* biomass), using likelihood function and chi‐square test. Interaction between treatment and day was included in the model. The Bonferroni *post‐hoc* test was used for pairwise comparisons at the 0.05 significance level. Uppercase letters indicate overall significant differences between treatments; lowercase letters indicate significant differences between each treatment of both time points at the 0.05 level. *n*, Number of plant replicates. Outliers are represented by circles (out).

We estimated plant resistance to the insect attackers by counting aphids and weighing caterpillars on plants exposed to single or dual attack. *B. brassicae* aphids performed best when feeding on plants that were simultaneously exposed to another attacker than on plants exposed to aphids only (Fig. 4). An overall effect of treatment was detected (Fig. 4; *P *<* *0.001): *B. brassicae* numbers were higher on plants exposed to dual attack by aphids plus *P. brassicae* (*P *=* *0.002) or aphids plus Xcr (*P *<* *0.001) than on plants infested with aphids only. *B. brassicae* were even more abundant on plants that were co‐infected with Xcr than on plants co‐infested with *P. brassicae* (Fig. 4, *P* < 0.001).

By contrast, *P. brassicae* caterpillars performed worse when feeding on plants that were simultaneously exposed to another attacker than on plants where the caterpillars were the only attacker (Fig. 4). An overall effect of treatment was detected (Fig. 4, *P* = 0.042). However, this effect was limited to plants that had been exposed to the treatments for 12 d (Fig. 4, interaction Treatment × Day, *P *=* *0.048). After 12 d of exposure of plants to single or dual attack, *P. brassicae* were heavier when caterpillars were the only attackers than on plants exposed to dual attack in the presence of *B. brassicae* (*P *=* *0.026).

### Effects of dual attack on plant indirect resistance

**Figure 5:**
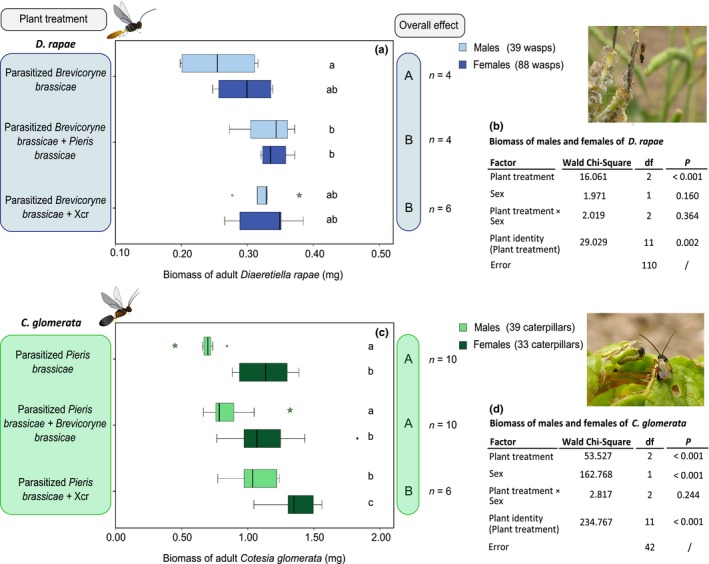
Fresh biomass of the parasitoid *Diaeretiella rapae* and of the parasitoid *Cotesia glomerata* developing in *Brevicoryne brassicae* aphids and *Pieris brassicae* caterpillars, respectively, reared on flowering *Brassica nigra* exposed to single or dual attack. (a) Fresh biomass of male and female *D. rapae* (median, 1^st^ and 3^rd^ quartiles, ± SD) and (c) of male and female *C. glomerata* (median, 1^st^ and 3^rd^ quartiles, ± SD) that emerged from their respective herbivorous hosts. Hosts of the parasitic wasps were reared on plants exposed to single or dual attack by *B. brassicae*,* P. brassicae* and/or *Xanthomonas campestris* pv *raphani* (Xcr). (b, d) Overall effects of the treatment were tested with a general linear model with normal distribution, using likelihood function and chi‐square test. The Bonferroni *post‐hoc* test was used for pairwise comparisons at the 0.05 significance level. Uppercase letters indicate overall significant differences between treatments; lowercase letters indicate significant differences between each treatment for males and females at the 0.05 level. *n*, Number of plant replicates; the number of wasps is given in parentheses. Outliers are represented by circles (out) and stars (far out).

We measured the biomass of male and female parasitoids, developmental time, and number of male and female parasitoids and used these parameters to assess the performance of parasitoids on plants exposed to single or simultaneous dual attack. Performance of the aphid parasitoid was affected by exposure of plants to dual attack, and males and females were differentially affected (Fig. 5). Biomass of *D. rapae* males was higher when the host aphids fed on plants that were simultaneously infested by *P. brassicae* caterpillars (*P *=* *0.046) than on plants infested by the aphids only or by the aphids plus bacteria. Biomass of female *D. rapae* was similar when the host aphid *B. brassicae* fed from plants exposed to the aphids only, and when the host fed from plants exposed to dual attack by either *P. brassicae* (*P *=* *0.297) or Xcr (*P *=* *1.000) (Fig. 5). Larvae of *D. rapae* developed more slowly when their aphid hosts fed from plants exposed to dual attack by aphids plus either *P. brassicae* (*P *<* *0.001) or Xcr (*P *<* *0.001) than on plants infested with their aphid hosts only (Fig. S3). Furthermore, numbers of male and female *D. rapae* that emerged from aphids were not affected by the treatments (Fig. S4).

By contrast, the caterpillar parasitoid, *C. glomerata*, performed better on plants exposed to dual attack by caterpillars plus bacteria than on plants exposed to caterpillars only or on plants exposed to caterpillars plus aphids (Fig. 5). Moreover, treatments affected males and females in a similar way. Irrespective of sex, *C. glomerata* were heavier when wasps emerged from host caterpillars that fed on plants exposed to dual attack by caterpillars plus Xcr than on plants infested with *P. brassicae* only (Fig. 5, males, *P *<* *0.001; females, *P *<* *0.001) or to dual attack by caterpillars plus *B. brassicae* (Fig. 5, males, *P *=* *0.002; females, *P *<* *0.001). Wasp biomass was similar for wasps that developed in host caterpillars feeding from plants simultaneously infested with *B. brassicae* and in host caterpillars feeding from plants infested with *P. brassicae* only (Fig. 5, males, *P *=* *0.170; females, *P *=* *1.000). Furthermore, the developmental time of *C. glomerata* was not influenced by dual attack either by *B. brassicae* or by Xcr (Fig. S3). Irrespective of sex, similar numbers of wasps emerged from host caterpillars that fed from plants infested only with the host *P. brassicae* and on plants exposed to dual attack by caterpillars plus either *B. brassicae* or Xcr (Fig. S4).

## Discussion

This study provides evidence for changes in the phytohormonal profile of the inflorescence upon exposure of flowering plants to single or simultaneous dual attack. Induction was mainly modulated by plant exposure to caterpillars, and was characteristic of flower tissues. Concentrations of jasmonates were especially high in dual‐attacked plants compared with plants exposed to single attack. Dual attack rendered plants more resistant to caterpillars but more susceptible to aphids. Furthermore, plant response to dual attack negatively affected the performance of parasitoids of the aphids, whereas it positively affected parasitoids of the caterpillars when compared with the single‐attack situation.

The phytohormonal profile of plants exposed to dual attack differed from that of plants exposed to single attack; higher concentrations of jasmonates were recorded in dual‐attacked plants than in single‐attacked plants. Our results demonstrate that jasmonates were enhanced in flower tissues, whereas no changes in SA and ABA concentrations were recorded following induction. We did not detect phytohormonal responses of plants to single attack by aphids or bacteria, suggesting that plants did not activate responses to these attackers under the conditions of our experiments. However, plants responded strongly to dual attack by caterpillars plus aphids and caterpillars plus bacteria, and to some extent, to aphids plus bacteria. Concentrations of biologically active jasmonates and their catabolites in flowers were higher when plants were exposed to dual attack by caterpillars plus bacteria than when exposed to single attack by caterpillars only, and different from the sum of the effects of both single attacks. This suggests a synergistic or additive effect of caterpillars and Xcr, as observed upon interactions with other microorganisms (Rodriguez‐Saona *et al*., 2010; Lazebnik *et al*., 2014), and this effect may strengthen resistance against both caterpillars and pathogens (Ton *et al*., 2002; Rostás *et al*., 2003; Lazebnik *et al*., 2014). Interestingly, high concentrations of jasmonates were also induced upon attack by caterpillars plus aphids.

Current knowledge on phytohormonal responses to insects and pathogens shows that aphids generally induce SA in plants at the vegetative stage (Heidel & Baldwin, 2004; Wu & Baldwin, 2010). Moreover, it is commonly accepted that the JA and SA pathways crosstalk, meaning that JA induction downregulates the SA pathway and SA induction downregulates the JA pathway (Kunkel & Brooks, 2002; Koornneef & Pieterse, 2008; Rodriguez‐Saona *et al*., 2010; Thaler *et al*., 2012), although some synergistic interactions are known as well (Kunkel & Brooks, 2002; Koornneef & Pieterse, 2008). In the present study on flowering plants, no SA induction was detected upon insect or pathogen attack, either in leaves or in flowers, despite the fact that a few hundred to a thousand aphids were feeding on the plants at the time points recorded. Interestingly, when compared with single attack by caterpillars, dual attack enhanced JA responses irrespective of the identity of the second attacker.

JA induction underlies resistance to chewing herbivores and occasionally to phloem feeders, although aphids mainly induce SA (Hansen & Halkier, 2005; Mewis *et al*., 2005; Mithöfer & Boland, 2012; Guo *et al*., 2013). Dual attack and the enhanced concentrations of jasmonates were reflected in stronger resistance of plants to caterpillars when compared with the caterpillar‐only attack situation, but compromised plant resistance to aphids. In fact, the development of aphids was not impaired, and these phloem feeders even benefited from dual attack despite the jasmonate induction in the flowers. There was no obvious competition for food between the two insect attackers during the experiment, and we think that direct competition is an unlikely explanation for the results observed. Plant exposure to *P. brassicae* caterpillars results in allocation of resources to flowers in *B. nigra* (Lucas‐Barbosa *et al*., 2017). Thus, we speculate that allocation of resources to flowers could facilitate the development of aphid colonies just below the flowers (Fig. 4), by increasing the nutritional quality of phloem in the inflorescence, and thus promoting aphid colony growth.

Plant responses to the attackers also affected the performance of parasitoids of the herbivorous insects. Parasitoids performed best when their host performed worse, and we expect that female parasitoids will preferably lay eggs in hosts where their offspring perform best. Our results show that female parasitoids of the aphids (*D. rapae*) developed more slowly on dual‐attacked plants, whereas parasitoids of the caterpillars (*C. glomerata*) were positively affected. Immune responses of the host insect can lead to encapsulation and killing of the parasitoid eggs, or negatively affect the development of the parasitoid larvae (Lackie, 1988). We observed that upon exposure to caterpillars and bacteria, plants exhibit high concentrations of jasmonates, which can lead to higher concentrations of resistance compounds. Thus, we speculate that the plant immune response possibly benefited the parasitoid by weakening the physiology of the host caterpillar, and the herbivore's ability to mount an effective immune response against parasitoids (Bukovinszky *et al*., 2009). We conclude that dual attack compromised important elements of plant direct and indirect resistance to aphids, but increased plant resistance to caterpillars. Based on this, we expect it to be advantageous for parasitoids to also respond to cues that can be associated with host plants that carry the best quality hosts, and that overall the complex phytohormone‐mediated interactions between multiple attackers can attenuate or enhance plant resistance depending on their feeding guild, with synergistic effects between key elements of plant direct and indirect defense.

The constitutive phytohormonal profile of leaves of flowering *B. nigra* plants is very different from that of flowers, and remarkably the phytohormonal profile of leaves remained unaffected when plants were exposed to single or dual attack, although true leaves of plants were directly exposed to eggs and caterpillars. Interestingly, jasmonates, their catabolites and, to some extent, ABA were present in higher concentrations in inflorescences than in leaves (see also Li *et al*., 2017), whereas SA and OPDA reached higher concentrations in leaves than in inflorescences. Plants responded to the attackers only with phytohormonal changes in flower tissues. To date, studies of plant responses to multiple attack have been made only for plants in the vegetative stage, and these showed that plant resistance can be negatively or positively affected when plants are exposed to more than one attacker (Soler *et al*., 2012; Lazebnik *et al*., 2014). Moreover, inducibility of resistance traits has been assumed to decrease with plant ontogeny (Diezel *et al*., 2011). Our data support the idea that inducibility of plant responses in flowering plants is rather canalized to flower tissues, where the phytohormonal profile changes in response to insect and pathogen attack. Indeed, recent studies have demonstrated that herbivore attack to leaves influences the volatile profile of flowers (Pareja *et al*., 2012; Bruinsma *et al*., 2014), and that resources can be allocated to flowers upon exposure to insect herbivores (Lucas‐Barbosa *et al*., 2017). For instance, folivory by *P. brassicae* caterpillars induced changes in the volatile blend of *B. nigra* flowers whereas the volatile emission of leaves did not change in response to attack (Bruinsma *et al*., 2014). It has been speculated that induction of phytohormones in inflorescences in response to attack could indirectly interfere with reproductive processes (Herms & Mattson, 1992; Strauss *et al*., 2002). Response to attack can modify flower chemistry and affect sugar composition of floral nectar (Euler & Baldwin, 1996; Strauss *et al*., 2004; Bruinsma *et al*., 2014), and affect flower–insect interactions, including changes in pollinator behavior (Lucas‐Barbosa *et al*., 2011; Bruinsma *et al*., 2014).

Our data show that the phytohormonal profile varied with time. To date, most data on phytohormonal responses to attack have been determined for short periods of induction, restricted to from a few hours to 3 d of induction (Stam *et al*., 2014), despite the fact that in natural conditions, plants are exposed to attackers throughout their development. The duration of exposure to the attackers and the amount of damage caused to the plants can provide a plausible explanation for the differences quantified over time. Indeed, plant responses can be affected by densities of attackers (Zhang *et al*., 2009; Kroes *et al*., 2015; Ponzio *et al*., 2016a), different larval stages can also induce different responses in plants (Erb *et al*., 2012) and ontogeny influences the phytohormonal profile of plant tissues (Du *et al*., 2008; Quintero & Bowers, 2011; Erbilgin & Colgan, 2012; Quintero *et al*., 2014). Phytohormonal analyses of leaves showed that concentrations were higher at day 12 than at day 8, and this may be the result of senescence of the leaves by day 12 (L. T. S. Chrétien, pers. obs.), supporting the hypothesis that plants redirect resources from leaves to the inflorescences upon attack, and activate resistance traits in flower tissues (Lucas‐Barbosa *et al*., 2013, 2016, 2017; Pashalidou *et al*., 2013; Lucas‐Barbosa, 2016), in accordance with the optimal defense theory (Cates & Rhoades, 1977). We speculate that plant responses to egg deposition on leaves, which typically induces SA, may have inhibited an early induction of JA in the inflorescence by the caterpillars when recorded at day 8, that is, 3 d after the caterpillars had hatched from the eggs, providing also a possible explanation of why higher phytohormonal concentrations were quantified at day 12 than at day 8 (Bruessow *et al*., 2010; Hilker & Fatouros, 2016).

Our study addressed for the first time, to our knowledge, inducible resistance of an annual plant in the flowering stage under multiple attack, and shows that dual attack promotes plant resistance to caterpillars, but compromises plant resistance to aphids. Caterpillars were the main inducers of plant responses, and the biologically active forms of JA were upregulated in flower tissues, overruling ABA and SA responses. We conclude that at the flowering stage of *B. nigra* plants the inducibility of defensive traits is redirected to the protection of reproductive tissues – something we expect to be typical of fast‐growing annual plants – and that under multiple attack, chewing herbivores are the main drivers of inducible plant resistance.

## Author contributions

L.T.S.C., D.L‐B. and M.D. planned and designed the study. L.T.S.C. collected most data and E.D. collected data on insect performance. A.D., J.G. and W.B. analyzed the phytohormones. L.T.S.C., A.D., W.B., D.L‐B., M.D. and D.G. analyzed and interpreted the data. L.T.S.C., D.L‐B., M.D. and D.G. wrote the manuscript.

## Supporting information

Please note: Wiley Blackwell are not responsible for the content or functionality of any Supporting Information supplied by the authors. Any queries (other than missing material) should be directed to the *New Phytologist* Central Office.

**Fig. S1** Concentration of active jasmonates and their catabolites (mean + SD) quantified in leaves and inflorescences of *Brassica nigra* plants exposed to single or dual attack for 8 or 12 d.
**Fig. S2** Concentration of abscisic acid (ABA), jasmonic acid (JA), *cis*‐(+)‐12‐oxophytodienoic acid (*cis*‐OPDA) and salicylic acid (SA) quantified in leaves and inflorescences (mean + SD) of *Brassica nigra* plants exposed to single or dual attack for 8 and 12 d.
**Fig. S3** Developmental time of the parasitoid *Diaeretiella rapae* and of the parasitoid *Cotesia glomerata* developing in *Brevicoryne brassicae* aphids and *Pieris brassicae* caterpillars, respectively, reared on flowering *Brassica nigra* plants exposed to single or dual attack.
**Fig. S4** Number of adult *Diaeretiella rapae* and adult *Cotesia glomerata* that emerged from *Brevicoryne brassicae* aphids and *Pieris brassicae* caterpillars, respectively, reared on flowering *Brassica nigra* plants exposed to single or dual attack.
**Table S1** Output of the generalized linear model for the effects of treatment, plant part and day (duration of exposure to the treatments) on the concentration of the jasmonic acid (JA)‐related phytohormones: the active forms (−)‐JA‐Ile and (+)‐7‐iso‐JA‐Ile, and of their catabolic forms 12‐OH‐JA, 12‐OH‐JA‐Ile and 12‐COOH‐JA‐Ile
**Table S2** Output of the generalized linear model for the effects of treatment, plant part and day (duration of exposure to the treatments) on the concentration of the phytohormones salicylic acid (SA), abscisic acid (ABA), jasmonic acid (JA) and *cis*‐(+)‐12‐oxophytodienoic acid (*cis*‐OPDA)
**Methods S1** Protocol for extraction and quantification of the phytohormones and their catabolites (adapted from Almeida Trapp *et al*., 2014).
