# Supplementary material for: Caterpillars induce jasmonates in flowers and alter plant responses to a second attacker
Source: New Phytol. 2017 Dec 5;217(3):1279–91. doi: 10.1111/nph.14904 (PMC5814890; doi:10.1111/nph.14904)
Supplement: Supplementary file 1 — Fig. S1 Concentration of active jasmonates and their catabolites (mean + SD) quantified in leaves and inflorescences of Brassica nigra plants exposed to single or dual attack for 8 or 12 d. Fig. S2 Concentration of abscisic acid (ABA), jasmonic acid (JA), cis‐(+)‐12‐oxophytodienoic acid (cis‐OPDA) and salicylic acid (SA) quantified in leaves and inflorescences (mean + SD) of Brassica nigra plants exposed to single or dual attack for 8 and 12 d. Fig. S3 Developmental time of the parasitoid Diaeretiella rapae and of the parasitoid Cotesia glomerata developing in Brevicoryne brassicae aphids and Pieris brassicae caterpillars, respectively, reared on flowering Brassica nigra plants exposed to single or dual attack. Fig. S4 Number of adult Diaeretiella rapae and adult Cotesia glomerata that emerged from Brevicoryne brassicae aphids and Pieris brassicae caterpillars, respectively, reared on flowering Brassica nigra plants exposed to single or dual attack. Table S1 Output of the generalized linear model for the effects of treatment, plant part and day (duration of exposure to the treatments) on the concentration of the jasmonic acid (JA)‐related phytohormones: the active forms (−)‐JA‐Ile and (+)‐7‐iso‐JA‐Ile, and of their catabolic forms 12‐OH‐JA, 12‐OH‐JA‐Ile and 12‐COOH‐JA‐Ile Table S2 Output of the generalized linear model for the effects of treatment, plant part and day (duration of exposure to the treatments) on the concentration of the phytohormones salicylic acid (SA), abscisic acid (ABA), jasmonic acid (JA) and cis‐(+)‐12‐oxophytodienoic acid (cis‐OPDA) Methods S1 Protocol for extraction and quantification of the phytohormones and their catabolites (adapted from Almeida Trapp et al., 2014). [file NPH-217-1279-s001.pdf]

Caterpillars induce jasmonates in flowers and alter plant responses to a second attacker

Lucille T.S. Chrétien[1,2,3], Anja David[4], Eirini Daikou[1], Wilhelm Boland[4], Jonathan Gershenzon[5], David Giron[2], Marcel Dicke[1], and Dani Lucas-Barbosa[1]

[1]Laboratory of Entomology, Wageningen University, Droevendaalsesteeg 1, Radix building, 6708PB Wageningen, The Netherlands

[2]Institut de Recherche sur la Biologie de l'Insecte (IRBI), UMR 7261, CNRS/Université François-Rabelais de Tours, Avenue Monge, Parc Grandmont, 37200 Tours, France

[3]Department of Biology, École Normale Supérieure de Lyon (ENS L), 46 Allée d'Italie, 69007 Lyon, France

[4]Department of Bioorganic Chemistry, Max Planck Institute for Chemical Ecology (MPI CE), Beutenberg Campus, Hans-Knoell-Strasse 8, D-07745 Jena, Germany

[5]Department of Biochemistry, Max Planck Institute for Chemical Ecology (MPI CE), Beutenberg Campus, Hans-Knoell-Strasse 8, D-07745 Jena, Germany

Corresponding author: Dani Lucas-Barbosa, dani.lucasbarbosa@wur.nl, 0031 0317 482388

Methods S1 Protocol for extraction and quantification of the phytohormones and their catabolites

Extraction of phytohormones was done by stirring 20 mg of ground freeze-dried plant material in 1.5 ml of methanol for 30 min, and then centrifuging it twice (at 14,000 rpm, for 10 min at 4 ºC) and combining the supernatants. The final methanolic crude extract was then evaporated (speed-vac at 30 ºC) and re-dissolved in 500 µl methanol. The following internal standards were added to the methanolic extract: 60 ng D_6-abscisic acid (D_6-ABA) (Santa Cruz Biotechnology, Santa Cruz, U.S.A.), 60 ng of D_6-jasmonic acid (D_6-JA) (HPC Standards GmbH, Cunnersdorf, Germany), 60 ng D_4-salicylic acid (D_6-SA) (Sigma-Aldrich, Merck KGaA, Darmstadt, Germany), and 12 ng of JA-$^{13}C_6$-isoleucine conjugate [JA-$^{13}C_6$-Ile]. To obtain JA-$^{13}C_6$-Ile, JA was conjugated to $^{13}C_6$-Ile (Sigma-Aldrich, Merck KGaA, Darmstadt, Germany) as described by Kramell *et al.* (Kramell *et al.*, 1988).

Resulting extracts were analysed by high performance liquid chromatography (Agilent 1200 HPLC system, Agilent technologies, Santa Clara, USA) coupled with a mass spectrometer (MS) (API 5000, Applied Biosystem, Foster city, USA) and equipped with a Turbospray ion source. Two µl of extracts was separated on a Zorbax Eclipse XDB-C18 column (50 x 4.6 mm, 1.8 µm, Agilent technologies, Santa Clara, USA). Two solvents formed the mobile phase: formic acid (0.05 %) in ultrapure water as solvent A, and acetonitrile as solvent B. The following gradient was used: 0-0.5 min, 5 % B; 0.5-9.5 min, 5-42 % B; 9.5-9.51 min, 42-100 % B; 9.51-12 min, 100 % B and 12.1-15 min, 5 % B. The flow rate was 1.1 ml min^{-1} and the column was kept at 25 ºC. In the MS, the liquid effluent was ionized by electrospray ionisation in a negative mode (-4500 eV). The turbo gas temperature was set at 700 ºC. Nebulizing gas was set at 60 psi, curtain gas at 25 psi, heating gas at 60 psi, and collision gas at 7 psi. The MS was run in multiple reaction monitoring (MRM) mode at *m/z* 263.0 to 153.2 (collision energy (CE) -22 V; declustering potential (DP) -35 V) for ABA; at *m/z* 269.0 to 159.2 (CE -22 V; DP -35 V) for D_6-ABA; at *m/z* 209.1 to 59.0 (CE -24 V; DP -35 V) for JA; at *m/z* 215.1 to 59.0 (CE -24 V; DP -35 V) for D_6-JA; at *m/z* 136.9 to 93.0 (CE -22 V; DP -35 V) for SA; at *m/z* 140.9 to 97.0 (CE -22 V; DP -35 V) for D_4-SA; at *m/z* 290.9 to 165.1 (CE -24 V; DP -45 V) for cis-OPDA, at *m/z* 322.2 to 130.1 (CE -30V; DP -50V) for JA-Ile conjugate; at *m/z* 328.2 to 136.1 (CE -30V; DP -50V) for JA-$^{13}C_6$-Ile conjugate; at *m/z* 338.2 to 130.1 (CE -

30V; DP -50V) for 12-hydroxy-jasmonoyl-isoleucine [12-OH-JA-Ile] conjugate; at *m/z* 352.2 to 130.1 (CE -30V; DP -50V) for 12-carboxyjasmonoyl-isoleucine [12-COOH-JA-Ile] conjugate; and at *m/z* 225.1 to 59.0 (CE -24V; DP -35V) for 12-hydroxy-jasmonate [12-OH-JA]. Phytohormones were quantified in ng g^{-1} of dry biomass (Analyst 1.5, Applied Biosystems, Foster city, USA) using their respective internal standards. The D_6-JA was used for the quantification of cis-OPDA with a response factor of 0.5, and for 12-OH-JA with a response factor of 1.0. 12-OH-JA-Ile conjugate and 12-COOH-JA-Ile conjugate were quantified using JA-$^{13}C_6$-Ile conjugate as internal standard applying a response factor of 1.0.

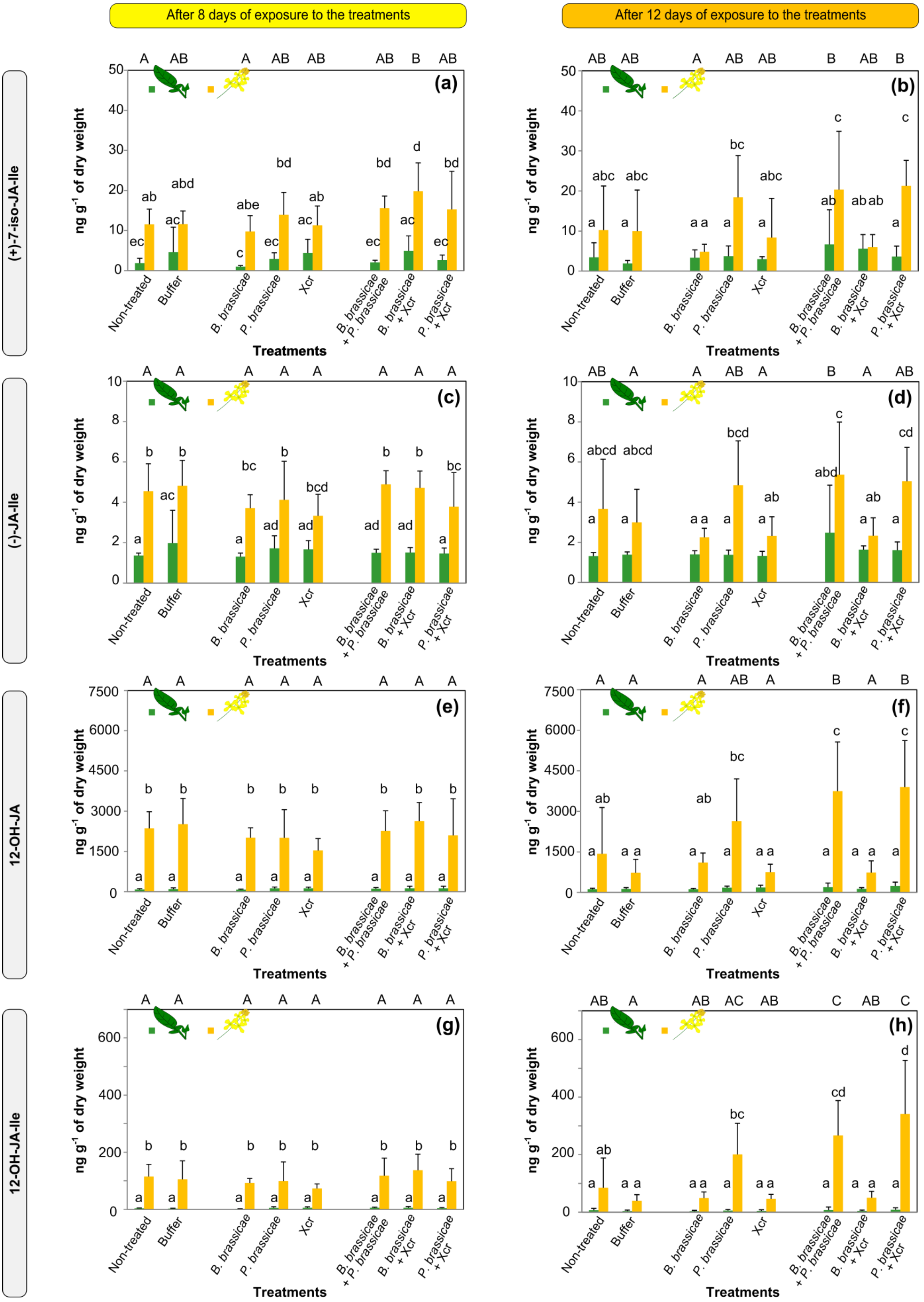
After 8 days of exposure to the treatments
After 12 days of exposure to the treatments
(+)-7-iso-JA-Ile
(-)-JA-Ile
12-OH-JA
12-OH-JA-Ile
(a)
(b)
(c)
(d)
(e)
(f)
(g)
(h)
ng g-1 of dry weight
Treatments
Non-treated
Buffer
B. brassicae
P. brassicae
Xcr
B. brassicae + P. brassicae
B. brassicae + Xcr
P. brassicae + Xcr

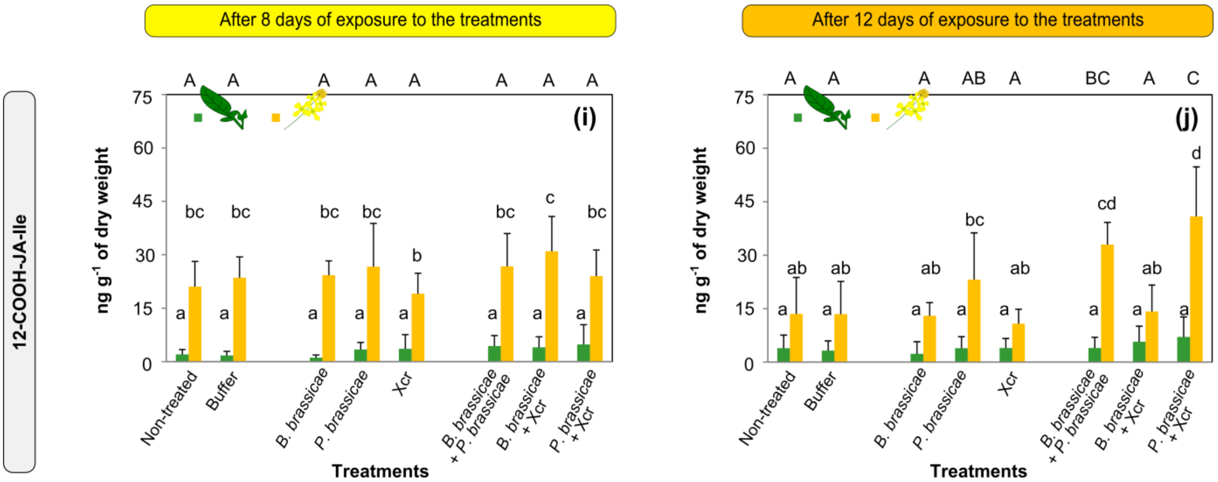

Fig. S1 Concentration of active jasmonates and their catabolites (mean + SD) quantified in leaves and inflorescences of *Brassica nigra* plants exposed to single or dual attack for 8 or 12 d.

Quantities (ng g^{-1} of plant dry weight) in leaves (green) and inflorescences (yellow) of the jasmonate-derived phytohormones: (+)-7-iso-jasmonoyl-L-isoleucine [(+)-7-iso-JA-Ile] at **(a)** day 8 and **(b)** day 12, and (-)-jasmonoyl-L-isoleucine [(-)-JA-Ile] at **(c)** day 8 and **(d)** day 12, and of the catabolic forms: 12-hydroxy-jasmonate [12-OH-JA] at **(e)** day 8 and **(f)** day 12, 12-hydroxy-jasmonoyl-isoleucine [12-OH-JA-Ile] at **(g)** day 8 and **(h)** day 12, 12-carboxy-jasmonoyl-isoleucine [12-COOH-JA-Ile] at **(i)** day 8 and **(j)** day 12, in plants that were nontreated, exposed to buffer, or exposed to single or dual attack by *Brevicoryne brassicae*, *Pieris brassicae*, and/or *Xanthomonas campestris* pv. *raphani* (Xcr). We had 6 replicates per treatment and time point. Uppercase letters indicate overall significant differences between treatments; lowercase letters indicate significant differences between each treatment for leaves and inflorescences at the 0.05 level.

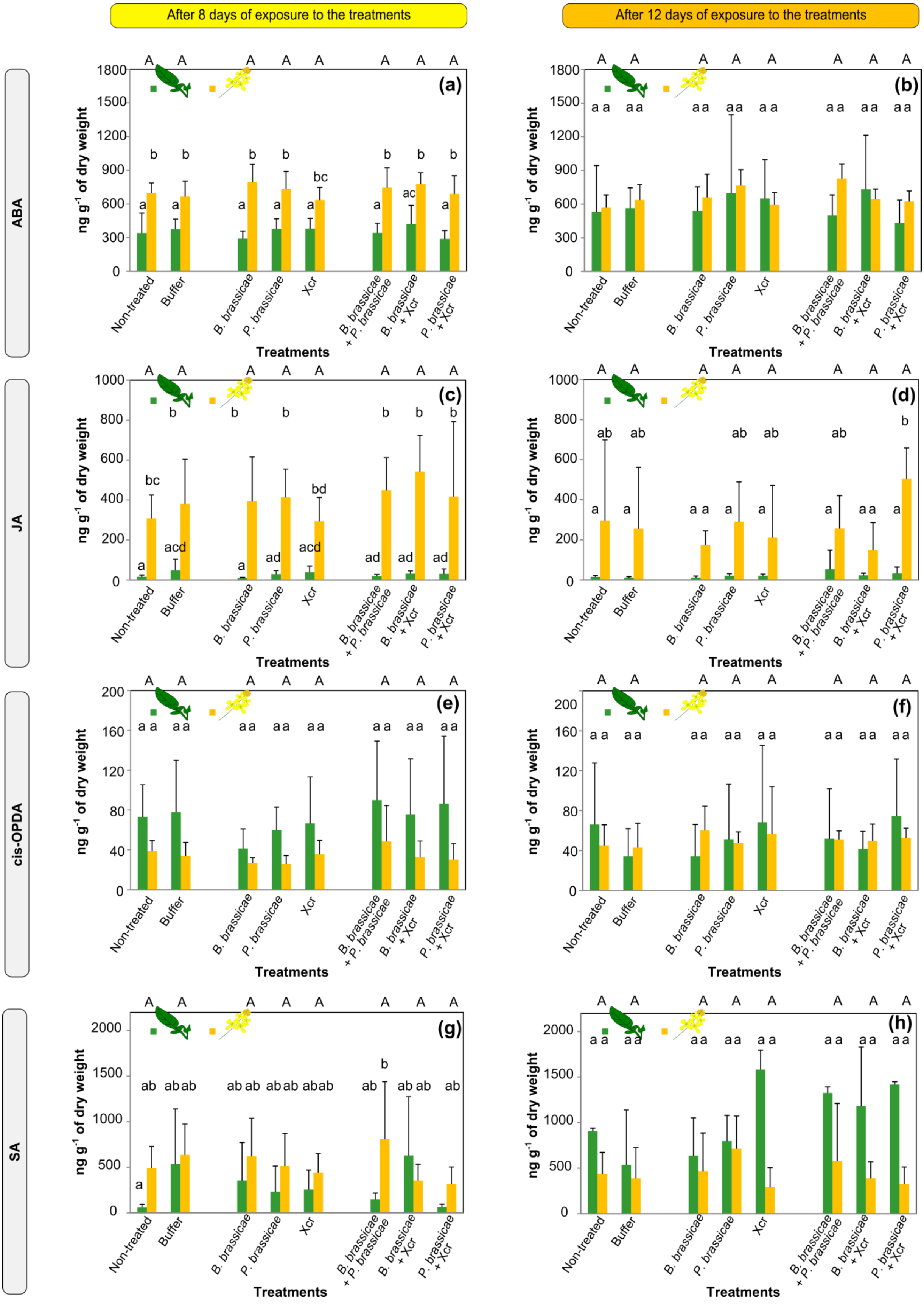

Fig. S2 Concentration of abscisic acid (ABA), jasmonic acid (JA), *cis*-(+)-12-oxophytodienoic acid (*cis*-OPDA) and salicylic acid (SA) quantified in leaves and inflorescences (mean + SD) of *Brassica nigra* plants exposed to single and dual attack for 8 and 12 d. Quantities (ng g^{-1} of dry weight) in leaves (green) and inflorescences (yellow) of the phytohormones: ABA at **(a)** day 8 and **(b)** day 12; JA at **(c)** day 8 and **(d)** day 12, *cis*-OPDA at **(e)** day 8 and **(f)** day 12; SA at **(g)** day 8 and **(h)** day 12, in plants that were nontreated, exposed to buffer, or exposed to single or dual attack by *Brevicoryne brassicae*, *Pieris brassicae*, and/or *Xanthomonas campestris* pv. *raphani* (Xcr). We had 6 replicates per treatment and time point. Uppercase letters indicate overall significant differences between treatments; lowercase letters indicate significant differences between each treatment for leaves and inflorescences at the 0.05 level.

Table S1 Output of the generalized linear model for the effects of treatment, plant part and day (duration of exposure to the treatments) on the concentration of the jasmonic acid (JA)-related phytohormones: the active forms (+)-7-iso-jasmonoyl-L-isoleucine [(+)-7-iso-JA-Ile] and (-)-jasmonoyl-L-isoleucine [(-)-JA-Ile], and of their catabolic forms 12-hydroxy-jasmonate [12-OH-JA], 12-hydroxy-jasmonoyl-isoleucine [12-OH-JA-Ile], 12-carboxy-jasmonoyl-isoleucine [12-COOH-JA-Ile]. We assessed compound concentration in leaves and inflorescences of flowering *Brassica nigra* plants that were exposed to single or dual attack for 8 or 12 d. Output of the analyses including both time points in the statistical model is shown on the left side. On the right side, the output for each of the time points is shown.

(+)-7-iso-JA-Ile

Overall

Factors	Wald Chi-Square	df	*P*
Treatment	29.113	7	< 0.001
Plant part	148.560	1	< 0.001
Day	0.046	1	0.830
Treatment*Plant part	20.323	7	0.005
Treatment*Day	19.421	7	0.007
Plant part*Day	1.699	1	0.192
Treatment*Plant part*Day	14.259	7	0.047

Day 8

Factors	Wald Chi-Square	df	*P*
Treatment	20.787	7	0.004
Plant part	163.381	1	< 0.001
Treatment*Plant part	11.333	7	0.125

Day 12

Factors	Wald Chi-Square	df	*P*
Treatment	25.611	7	< 0.001
Plant part	41.058	1	< 0.001
Treatment*Plant part	19.592	7	0.007

(-)-JA-Ile

Overall

Factors	Wald Chi-Square	df	*P*
Treatment	29.34	7	< 0.001
Plant part	216.308	1	< 0.001
Day	3.908	1	0.048
Treatment*Plant part	15.373	7	0.032
Treatment*Day	20.146	7	0.005
Plant part*Day	4.046	1	0.044
Treatment*Plant part*Day	13.090	7	0.070

Day 8

Factors	Wald Chi-Square	df	*P*
Treatment	11.116	7	0.134
Plant part	206.233	1	< 0.001
Treatment*Plant part	8.615	7	0.281

Day 12

Factors	Wald Chi-Square	df	*P*
Treatment	31.726	7	< 0.001
Plant part	60.949	1	< 0.001
Treatment*Plant part	17.110	7	0.017

12-OH-JA

Overall

Factors	Wald Chi-Square	df	*P*
Treatment	44.288	7	< 0.001
Plant part	368.557	1	< 0.001
Day	1.550	1	0.213
Treatment*Plant part	40.043	7	< 0.001
Treatment*Day	47.403	7	< 0.001
Plant part*Day	3.110	1	0.078
Treatment*Plant part*Day	43.510	7	< 0.001

Day 8

Factors	Wald Chi-Square	df	*P*
Treatment	8.202	7	0.315
Plant part	349.145	1	<0.001
Treatment*Plant part	8.756	7	0.271

Day 12

Factors	Wald Chi-Square	df	*P*
Treatment	63.125	7	<0.001
Plant part	110.69	1	<0.001
Treatment*Plant part	56.934	7	<0.001

12-OH-JA-Ile

Overall

Factors	Wald Chi-Square	df	*P*
Treatment	64.975	7	< 0.001
Plant part	260.717	1	< 0.001
Day	4.996	1	0.025
Treatment*Plant part	61.011	7	< 0.001
Treatment*Day	62.301	7	< 0.001
Plant part*Day	4.071	1	0.044
Treatment*Plant part*Day	60.039	7	< 0.001

Day 8

Factors	Wald Chi-Square	df	*P*
Treatment	7.713	7	0.359
Plant part	234.939	1	<0.001
Treatment*Plant part	7.258	7	0.403

Day 12

Factors	Wald Chi-Square	df	*P*
Treatment	78.722	7	<0.001
Plant part	104.735	1	<0.001
Treatment*Plant part	74.892	7	<0.001

12-COOH-JA-Ile

Overall

Factors	Wald Chi-Square	df	*P*
Treatment	61.905	7	< 0.001
Plant part	469.932	1	< 0.001
Day	3.259	1	0.071
Treatment*Plant part	34.191	7	< 0.001
Treatment*Day	34.489	7	< 0.001
Plant part*Day	9.861	1	0.002
Treatment*Plant part*Day	34.950	7	< 0.001

Day 8

Factors	Wald Chi-Square	df	*P*
Treatment	12.679	7	0.080
Plant part	359.996	1	<0.001
Treatment*Plant part	8.225	7	0.313

Day 12

Factors	Wald Chi-Square	df	*P*
Treatment	74.745	7	<0.001
Plant part	150.127	1	<0.001
Treatment*Plant part	54.262	7	<0.001

Table S2 Output of the generalized linear model for the effects of treatment, plant part and day (duration of exposure to the treatments) on the concentration of the phytohormones: salicylic acid (SA), abscisic acid (ABA), jasmonic acid (JA), and *cis*-(+)-12-oxophytodienoic acid (*cis*-OPDA). We assessed compound concentration in leaves and inflorescences of flowering *Brassica nigra* plants that were exposed to single or dual attack for 8 or 12 d. Output of the analyses including both time points in the statistical model is shown on the left side. On the right side, the output for each of the time points is shown.

ABA

Overall

Factors	Wald Chi-Square	df	*P*
Treatment	9.813	7	0.199
Plant part	59.929	1	< 0.001
Day	9.267	1	0.002
Treatment*Plant part	8.787	7	0.268
Treatment*Day	2.392	7	0.935
Plant part*Day	23.332	1	< 0.001
Treatment*Plant part*Day	3.357	7	0.850

Day 8

Factors	Wald Chi-Square	df	*P*
Treatment	7.205	7	0.408
Plant part	240.084	1	< 0.001
Treatment*Plant part	9.121	7	0.244

Day 12

Factors	Wald Chi-Square	df	*P*
Treatment	5.885	7	0.553
Plant part	2.536	1	0.111
Treatment*Plant part	5.471	7	0.603

JA

Overall

Factors	Wald Chi-Square	df	*P*
Treatment	8.948	7	0.256
Plant part	218.377	1	< 0.001
Day	10.985	1	0.001
Treatment*Plant part	10.470	7	0.163
Treatment*Day	7.075	7	0.421
Plant part*Day	9.512	1	0.002
Treatment*Plant part*Day	10.687	7	0.153

Day 8

Factors	Wald Chi-Square	df	*P*
Treatment	7.184	7	0.410
Plant part	181.623	1	< 0.001
Treatment*Plant part	7.311	7	0.397

Day 12

Factors	Wald Chi-Square	df	*P*
Treatment	11.687	7	0.111
Plant part	60.951	1	< 0.001
Treatment*Plant part	10.111	7	0.182

cis-OPDA

Overall

Factors	Wald Chi-Square	df	*P*
Treatment	7.594	7	0.370
Plant part	15.660	1	< 0.001
Day	0.033	1	0.856
Treatment*Plant part	5.519	7	0.597
Treatment*Day	5.322	7	0.621
Plant part*Day	12.673	1	< 0.001
Treatment*Plant part*Day	1.749	7	0.972

Day 8

Factors	Wald Chi-Square	df	*P*
Treatment	8.761	7	0.270
Plant part	31.468	1	<0.001
Treatment*Plant part	2.903	7	0.894

Day 12

Factors	Wald Chi-Square	df	*P*
Treatment	4.583	7	0.711
Plant part	0.072	1	0.789
Treatment*Plant part	4.230	7	0.753

SA

Overall

Factors	Wald Chi-Square	df	*P*
Treatment	3.885	7	0.793
Plant part	5.431	1	0.020
Day	19.907	1	< 0.001
Treatment*Plant part	10.932	7	0.142
Treatment*Day	10.344	7	0.170
Plant part*Day	29.203	1	< 0.001
Treatment*Plant part*Day	9.550	7	0.216

Day 8

Factors	Wald Chi-Square	df	*P*
Treatment	13.316	7	0.065
Plant part	12.605	1	<0.001
Treatment*Plant part	14.090	7	0.05

Day 12

Factors	Wald Chi-Square	df	*P*
Treatment	5.685	7	0.577
Plant part	18.404	1	<0.001
Treatment*Plant part	9.354	7	0.228

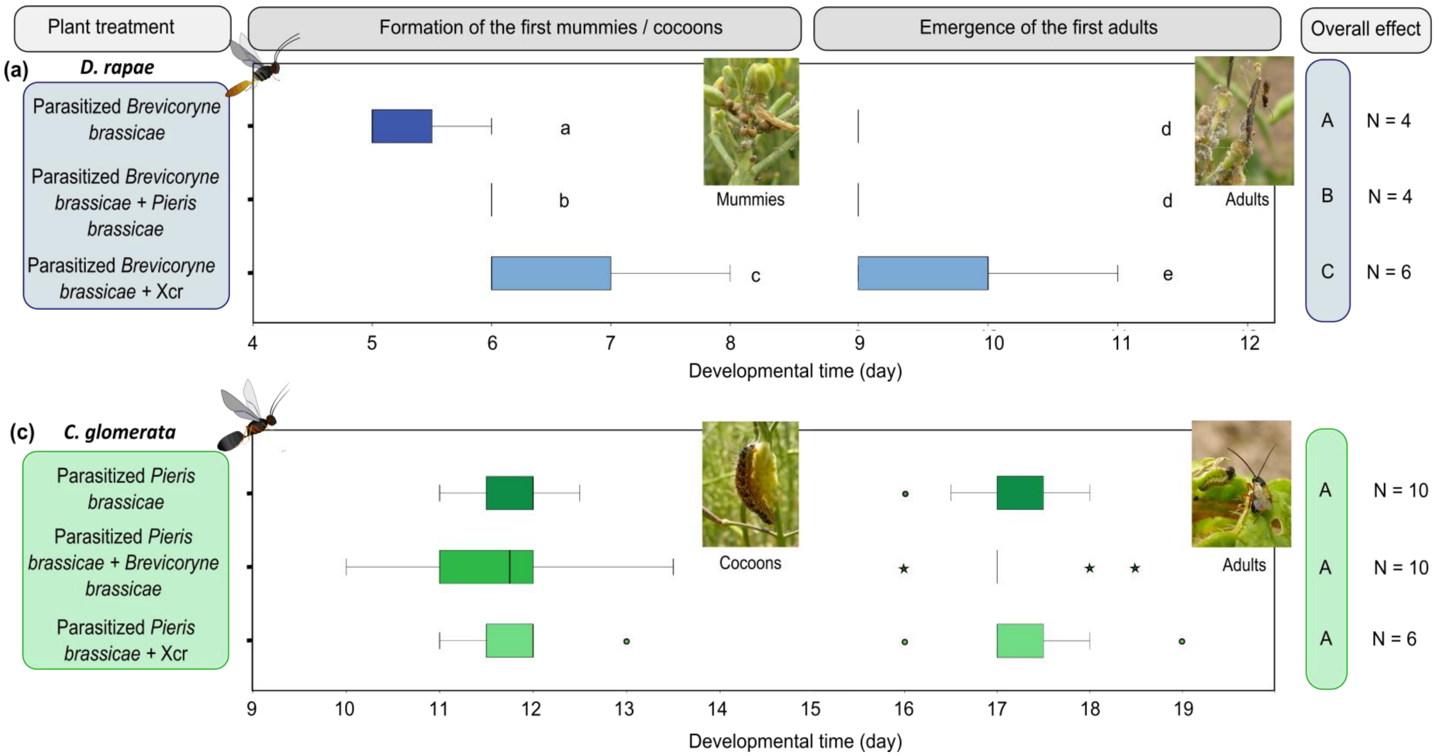

(b)

Developmental time of *D. rapae*

Factors	Wald Chi-Square	df	P
Plant Treatment	107.634	2	< 0.001
Developmental stage	1394.995	1	< 0.001
Plant treatment * Developmental stage	23.988	2	< 0.001
Plant identity (Plant treatment)	99.645	11	< 0.001
Error		11	/

(d)

Developmental time of *C. glomerata*

Factors	Wald Chi-Square	df	P
Plant treatment	3.215	2	0.200
Developmental stage	3662.661	1	< 0.001
Plant treatment * Developmental stage	0.929	2	0.628
Plant identity (Plant treatment)	213.044	24	< 0.001
Error		50	/

Fig. S3 Developmental time of the parasitoid *Diaeretiella rapae* and of the parasitoid *Cotesia glomerata* developing in *Brevicoryne brassicae* aphids and *Pieris brassicae* caterpillars, respectively, reared on flowering *Brassica nigra* plants exposed to single or dual attack.
(a) Developmental time of males and females *D. rapae* (median, 1st and 3rd quartiles, ± SD) and **(c)** of males and females *C. glomerata (*median, 1st and 3rd quartiles, ± SD) that developed in and emerged from their respective herbivorous hosts. Hosts of the parasitic wasps were reared on plants exposed to single or dual attack by *B. brassicae*, *P. brassicae*, and/or *Xanthomonas campestris* pv. *raphani* (Xcr). **(b, d)** Statistics; overall effects of the treatment were tested with a general linear model with a normal distribution using likelihood function and chi-square test. Interaction between plant treatment and developmental stage was included in the model. The Bonferroni *post-hoc* test was used for pairwise comparisons at the 0.05 significance level. Uppercase letters indicate overall significant differences between treatments, lowercase letters indicate significant differences between each treatment for males and females at the 0.05 level. N, Number of plant replicates. Outliers are represented by circles (out) and stars (far out).

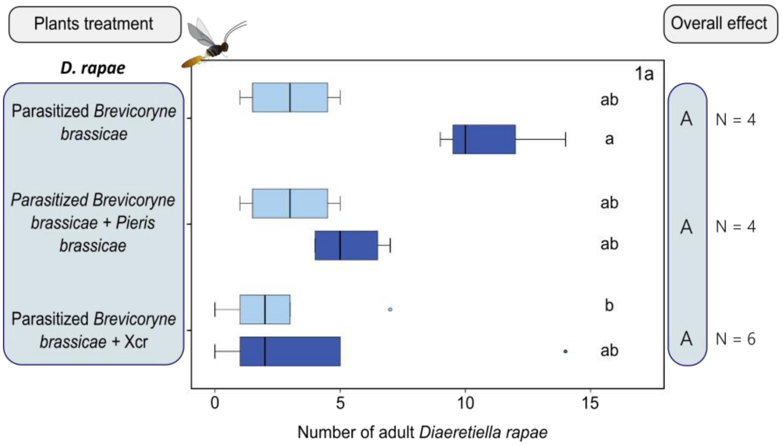

Males
Females

1b

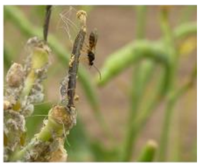

Number of males and females of *D. rapae*

Factors	Wald Chi-Square	df	P
Plant treatment	3.543	2	0.170
Sex	5.842	1	0.016
Plant treatment * Sex	1.356	2	0.508
Plant identity (Plant treatment)	6.547	11	0.835
Error		11	/

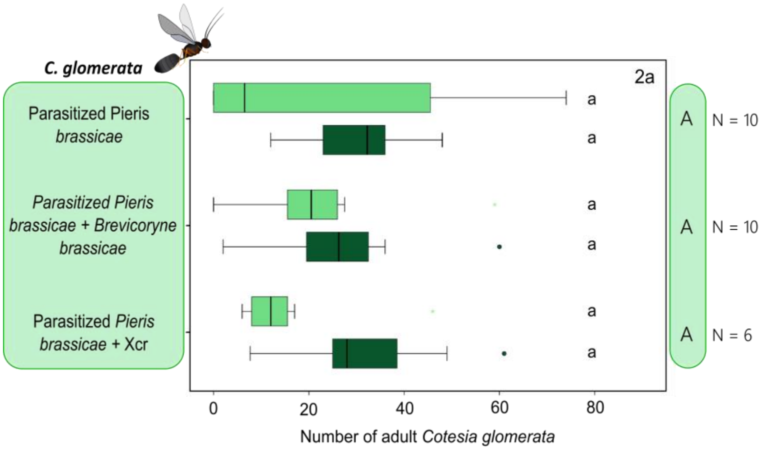

Males
Females

2b

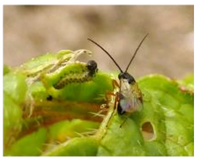

Number of males and females of *C. glomerata*

Factors	Wald Chi-Square	df	P
Plant treatment	2.059	2	0.357
Sex	2.586	1	0.108
Plant treatment * Sex	3.899	2	0.142
Plant identity (Plant treatment)	52.280	24	0.001
Error		50	/

Fig. S4 Number of adults *Diaeretiella rapae* and of adults *Cotesia glomerata* that emerged from *Brevicoryne brassicae* aphids and *Pieris brassicae* caterpillars, respectively, reared on flowering *Brassica nigra* plants exposed to single or dual attack.
(a) Number of males and females *D. rapae* (median, 1st and 3rd quartiles, ± SD) and **(a)** of males and females *C. glomerata* (median, 1st and 3rd quartiles, ± SD) that emerged from their respective herbivorous hosts. Hosts of the parasitic wasps were reared on plants exposed to single or simultaneous dual attack by *B. brassicae*, *P. brassicae*, and/or *Xanthomonas campestris* pv. *raphani* (Xcr). (**b, d)** Statistics; overall effects of the treatment were tested with a general linear model with a poisson distribution using likelihood function and chi-square test. The Bonferroni *post-hoc* test was used for pairwise comparisons at the 0.05 significance level. Uppercase letters indicate overall significant differences between treatments, lowercase letters indicate significant differences between each treatment for males and females at the 0.05 level. N, Number of plant replicates. Outliers are represented by circles (out) and stars (far out).

References

Kramell R, Schmidt J, Schneider G, Sembdner G, Schreiber K. 1988. Synthesis of n-(jasmonoyl)amino acid conjugates. *Tetrahedron* **44**(18): 5791-5807.
